# The Acute Side Effects of Bright Light Therapy: A Placebo-Controlled Investigation

**DOI:** 10.1371/journal.pone.0075893

**Published:** 2013-09-24

**Authors:** Yevgeny Botanov, Stephen S. Ilardi

**Affiliations:** Department of Psychology, University of Kansas, Lawrence, Kansas, United States of America; University of Pennsylvania School of Medicine, United States of America

## Abstract

Despite the emergence of numerous clinical and non-clinical applications of bright light therapy (LT) in recent decades, the prevalence and severity of LT side effects have not yet been fully explicated. A few adverse LT effects—headache, eye strain, irritability, and nausea—have been consistently reported among depressed individuals and other psychiatric cohorts, but there exists little published evidence regarding LT side effects in non-clinical populations, who often undergo LT treatment of considerably briefer duration. Accordingly, in the present study we examined, in a randomized sample of healthy young adults, the acute side effects of exposure to a single 30-minute session of bright white light (10,000 lux) versus dim red light (< 500 lux). Across a broad range of potential side effects, repeated-measures analyses of variance revealed no significant group-by-time (Pre, Post) interactions. In other words, bright light exposure was not associated with a significantly higher incidence of any reported side effect than was the placebo control condition. Nevertheless, small but statistically significant increases in both eye strain and blurred vision were observed among *both* the LT and control groups. Overall, these results suggest that the relatively common occurrence of adverse side effects observed in the extant LT literature may not fully extend to non-clinical populations, especially for healthy young adults undergoing LT for a brief duration.

## Introduction

Although medical curiosity about the salubrious benefits of sunlight exposure can be traced to antiquity [1,2], contemporary clinical interest was ignited by the 20^th^-century discovery of a direct link between light exposure and circadian melatonin production [3,4]. An obvious initial candidate for bright light therapy (LT) was *seasonal affective disorder* [5] – a form of depressive illness typically triggered by light deprivation during the short, cold days of winter – and the intervention has proven to be efficacious across a large number of randomized controlled trials [6-8]. In fact, the efficacy of LT has now been supported across a range of other mood disorders, including non-seasonal depression [9,10,6], bipolar disorder [11,12], antepartum and postpartum depression [13,14], and premenstrual dysphoric disorder [15,16].

Light therapy (LT) has also been successfully applied to the treatment of sleep disorders [17-20], as well as circadian phase sleep disorders associated with jet lag [21,22] and shift work [23,24]. More recently, LT has shown promise as an intervention for obsessive-compulsive symptoms [25], behavioral disturbances and functioning in dementia and Alzheimer’s disease [26,27], primary and secondary features of Parkinson’s disease [28], attention deficit hyperactivity disorder [29], seasonal variations in eating disturbances associated with bulimia nervosa [30,31], and some symptoms of chronic anorexia [32].

The most prominently promoted putative mechanism underlying LT’s therapeutic effect is the inhibition and shifting of melatonin production in the brain’s pineal gland, which may in turn induce a therapeutic alteration of dysregulated circadian rhythms [4]. In other words, LT can trigger a phase-shifting of the brain’s circadian clock, with the strongest therapeutic effect occurring in individuals with seasonal onset depression [6]. But retinal bright light exposure is also capable of altering neurotransmitter function in a number of cerebral circuits. For example, such exposure directly influences serotonin turnover in the brain, with the lowest rate of turnover in the winter, and more rapid turnover with exposure to increased luminosity [33]. Likewise, LT appears to enhance dopamenergic transmission in some brain regions [34,35].

Despite the established therapeutic potential of LT in seasonal depression and potential usefulness across a wide array of application, considerably less is known about the side effects and tolerability of LT. While a handful of landmark studies (e.g., [36-39]) have concluded that headache and eye/vision difficulties are the most common side effects of LT among mood-disordered patients, we can identify only one published study that has reported on the adverse effects of LT while employing a placebo control [40]. Interestingly, this study observed no significant differences in side effects between the LT and placebo groups, although it is worth noting that the dosage of light was considerably lower than that most commonly prescribed in contemporary practice. One additional study [41] examined the side effects of a head-mounted visor that delivered white light at illuminance levels of 60 lux, 600 lux, or 3500 lux, respectively. We do not regard this as an adequate test of LT effects, however, in as much as it utilized a non-standard light delivery method (visor) with considerably less empirical support.

Perhaps most importantly, to our knowledge there exist no published studies regarding the acute side effects of LT among non-psychiatric populations. On the contrary, most of what we know about LT side effects comes from the study of mood-disordered individuals, who are known to exhibit increased attentional vigilance for negative information [42], along with an increased propensity to interpret physiological changes as aversive [43], and increased sensation of anticipatory pain [44]. Such depressive interpretive biases could conceivably lead to the over-reporting of adverse effects while undergoing interventions such as LT. Accordingly, the examination of LT side effects among non-psychiatric individuals has the potential to be of some conceptual and applied utility. Moreover, we are aware of no placebo-controlled examinations of LT side effects utilizing the most commonly prescribed dosage and duration of LT -10,000 lux for 30 minutes [45].

The purpose of the present investigation, therefore, is to address the aforementioned gaps in the extant LT literature. Specifically, we evaluated the side effect profile of healthy young adults undergoing a 30-minute session of standard LT in comparison with that of a placebo control condition (of less than 500 lux red light). Since most LT side effects are known to emerge during the initial session and to diminish with repeated exposures [38], the present study evaluated the side effects associated with only one session at the currently prescribed dose and illuminance (10,000 lux).

## Materials and Methods

### Ethics Statement

Participants provided written informed consent, and the study was approved by the University of Kansas Institutional Review Board.

### Participants

All study participants were recruited from an introductory psychology class at a large university in the midwestern United States. Exclusion criteria included only a self-reported history of bipolar disorder or retinal light sensitivity. However, nine participants were also excluded from the final study analyses because they reported a history of major depressive disorder. An additional six participants endorsed *every item* on a side effect questionnaire administered prior to the study’s light exposure manipulation, as this presumably indicated either the presence of clinically significant symptomatology or a misunderstanding of study instructions and were excluded from analyses.

### Procedure

Artificial bright light was provided by a Sunlight Jr. light box (The Sunbox Company, Gaithersburg, MD). The Sunlight Jr. is a triangular box (14.5" Tall x 7" Wide (Face) x 6″ Sides) that emits a full spectrum of light and employs a spectrally transparent prismatic diffuser to block ultraviolet rays. At a distance of 14 inches, the box emits an illuminance of 10,000 lux. A red filter was positioned over the prismatic diffuser to filter all but red light and thereby to reduce the illuminance to approximately 450 lux at a distance of 14 inches.

After the consent process, participants completed a brief assessment battery to determine study eligibility, after which eligible participants were randomly assigned, via a computer generated random ordered list, either to the bright white light condition (10,000 lux) or the low-level red light condition (approximately 450 lux). In both light conditions, individuals were seated alone at a table in a room with a light box positioned 14 inches from their eyes, above their head, and facing them at a 45 degree angle. To maintain a naturalistic balance between the experimental light sources and the ambient room light, the experiment room’s fluorescent overhead lighting was dimmed to 50 lux.

During the 30-minute light exposure, participants were instructed to read popular culture and/or current events magazines provided by the experimenters, and to maintain gaze forward, i.e., in the direction of the overhead light box. An experimenter unobtrusively monitored adherence to the procedural instructions throughout the session, but did not interact with the participants in any other capacity. After the 30-minute exposure, participants completed a final assessment battery. All sessions occurred between 8:00 a.m. and 11:00 a.m. in a room with no natural light sources. Data collection began in November 2010 and ended in mid-December 2011.

### Measures

The Toronto Side Effect Scale (TSES) is a 32-item clinician-rated instrument that measures the occurrence of adverse treatment events [46]; it assesses both frequency (never – everyday) and severity (no trouble – extreme trouble) on a 5-point Likert-type scale. The TSES is well-suited to modification in line with specific treatment study goals (e.g. [47,48]). Accordingly, TSES was modified in the present investigation by excluding 15 items that measure side effects of psychotropic medication inapplicable to LT (e.g. sexual dysfunction, weight loss/gain), along with the addition of two items to reflect symptoms specifically relevant to LT (e.g. irritability, eye strain). The aforementioned modifications yielded a 19-item self-report measure of physiological symptoms of potential relevance to LT. Additionally, the TSES frequency scale was slightly modified to present a binary choice regarding the presence or absence of each side effect, while the measure’s 5-point side effect severity scale was preserved but relabeled to facilitate greater participant comprehension (mild – severe). Finally, a total side effect intensity score was calculated as the sum total of severity ratings across the measure’s 19 items.

### Data Analysis

All data analyses were conducted using PASW Statistics 18, Release Version 18.0.0 (SPSS, Inc., 2009, Chicago, IL, www.spss.com). First, the presence of potentially confounding between-group differences on demographic variables (gender, age), recent outdoor light exposure, and relevant clinical characteristics (sleep quantity and physiological symptoms present prior to LT) were tested by means of univariate analyses of variance. A within-subjects, repeated measures, 2x2 factorial design using time (pre, post) by condition (bright light, red light) was used to analyze potential side effects. Specifically, main effects of time (pre, post) and light condition (bright white, dim red), as well as a time-by-condition interaction effect, were analyzed using a repeated measures analysis of variance (ANOVA) for total side effect intensity and severity of the most commonly reported side effects of LT as measured by the TSES. Effect sizes were calculated using Cohen’s *d* [49]. To limit the potential inflation of experimentwise Type I error due to multiple significance tests, an alpha level of .01 was utilized in all analyses.

A post hoc analysis was also conducted to examine seasonal variability in report of side effects. A within-subjects, repeated measures, 2x2x4 factorial design – time (pre, post) by condition (bright light, red light) by season (summer, fall, winter, spring) – was used to analyze changes in each side effect. Similarly, an alpha level of .01 was utilized to limit inflation of Type I error.

## Results

Two hundred and thirteen undergraduate students (56.3% female) met criteria for the study and were included in the final analyses. The age of participants ranged from 18 to 32 (*M* = 19, *SD* = 2) who slept an average of 6.8 hours (SD = 1.5) the night before the study. One hundred and twelve (52.6%) participants were randomized into the red light control group, and 101 into the bright light group. The percentage of participants in each group did not differ significantly by gender, X^2^ (1, N = 213) = .06, *p* = .80. Likewise, univariate analyses of variance revealed no significant preexisting differences in baseline characteristics between participants in the two experimental conditions on age (*t*[211] = -.93, *p* = .35), hours of sleep (*t*[211] = .31, *p* = .76), or time spent outdoors prior to the experiment (*t*[209] = .01, *p* = .99). Similarly, no pre-treatment differences were seen for nausea (*t*[21] = .18, *p* = .86), headache (*t*[211] = -.03, *p* = .98), blurred vision (*t*[211] = 1.28, *p* = .20) or eye strain (*t*[211] = .16, *p* = .87). Table 1 presents the severity of side effects at pre-treatment and post-treatment for each light condition.

**Table 1:** Average Side Effect Intensity for Dim Red and Bright White Light at Pre-Treatment and Post-Treatment.

	Pre-Treatment				Post-Treatment			
	Red		White		Red		White	
Symptom	Mean	SD	Mean	SD	Mean	SD	Mean	SD
Nervousness	.5	(.8)	.6	(1.1)	.1	(.5)	.3	(.8)
Agitation	.1	(.3)	.3	(.7)	.2	(.7)	.2	(.6)
Tremor or shakiness	.1	(.5)	.1	(.5)	.1	(.5)	.1	(.3)
Muscle twitching	.2	(.6)	.1	(.4)	.1	(.2)	.1	(.3)
Abdominal pain	.2	(.6)	.1	(.4)	.1	(.4)	.1	(.3)
Upset stomach	.2	(.7)	.3	(.7)	.1	(.5)	.2	(.6)
Nausea	.1	(.4)	.1	(.4)	.1	(.4)	0	(.2)
Weakness or fatigue	.5	(1.0)	.7	(1.2)	.7	(1.2)	.7	(1.3)
General dizziness	.1	(.4)	.1	(.4)	.2	(.5)	.2	(.6)
Daytime drowsiness	.6	(1.1)	1.0	(1.4)	.8	(1.2)	.9	(1.3)
Sweating	.1	(.5)	.3	(.8)	0	(.1)	0	(.2)
Flushing	0	(.3)	.1	(.4)	0	(.1)	0	(.3)
Headache	.4	(1.0)	.4	(.9)	.5	(.9)	.5	(1.0)
Blurred vision	.1	(.5)	.1	(.3)	.2	(.5)	.2	(.5)
Eye strain	.1	(.5)	.1	(.4)	.5	(1.0)	.4	(.9)
Dry mouth	.1	(.5)	.3	(.7)	.2	(.6)	.2	(.6)
Irritability	.2	(.6)	.2	(.6)	.2	(.7)	.1	(.5)
Restless energy	.2	(.6)	.2	(.6)	.2	(.7)	.2	(.7)
Total side effect intensity	3.7	(5.3)	4.7	(6.1)	4.2	(5.6)	4.4	(5.3)

Analysis of variance (ANOVA) showed a significant main effect of time on eye strain, *F*(1, 211) = 32.36, *p* < .001, d = .47, and blurred vision, *F*(1, 211) = 7.60, *p* = .006, d = .19. Across both groups, the average post-treatment eye strain severity (*M* = .4, *SD* = 1.0) was greater than pre-treatment severity (*M* = .1, *SD* = .5); likewise, average post-treatment blurred vision severity (*M* = .2, *SD* = .5) was greater than the pre-treatment mean (*M* = .1, *SD* = .4). A significant main effect of time was also observed for nervousness, *F*(1, 209) = 40.01, *p* < .001, muscle twitching, *F*(1, 211) = 7.94, *p* = .005, and sweating, *F*(1, 210) = 14.27, *p* < .001, with severity scores *lower* at post-treatment on all three variables (Table 1). There was no significant main effect for headache, *F*(1, 211) = 2.41, *p* = .12, or nausea, *F*(1, 211) = .61, *p* = .43. Similarly, there was no significant main effect for total side effect intensity, *F*(1, 211) = .07, *p* = .80. There were no significant time-by-condition interaction effects and post hoc testing showed no significant effects of season.

## Discussion

The present investigation represents the first placebo-controlled examination of bright light side effects at the standard recommended dosage -10,000 lux for 30 minutes [45] – in a non-clinical adult sample. We observed no significant between-group differences (bright light versus red light control) in the occurrence of any side effects, including eye strain, blurred vision, nausea and headache (see Table 2). Likewise, we found no significant pre-post increase in the study’s composite side effect scale as a function of bright light exposure.

**Table 2:** Percentage of Side Effects Reported for Dim Red and Bright White Light at Pre-Treatment and Post-Treatment.

	Pre-Treatment		Post-Treatment	
	Red	White	Red	White
Symptom	(n = 112)	(n = 101)	(n = 112)	(n = 101)
Nervousness	36	34	12	17
Agitation	6	14	13	11
Tremor or shakiness	10	7	6	7
Muscle twitching	12	8	6	5
Abdominal pain	12	4	8	4
Upset stomach	12	14	11	11
Nausea	5	5	7	2
Weakness or fatigue	29	32	39	31
General dizziness	6	5	13	9
Daytime drowsiness	33	42	45	44
Sweating	10	14	2	4
Flushing	2	5	1	1
Headache	18	22	32	27
Blurred vision	8	4	13	13
Eye strain	6	5	26	22
Dry mouth	9	15	13	14
Irritability	11	10	13	10
Restless energy	12	11	14	14

Our findings run somewhat counter to those of several previous light therapy (LT) investigations (e.g., [36-38]). This discrepancy may be due to any of the three principal methodological differences from the aforementioned studies – a single exposure, comparison to a dim light control, and a sample of healthy young adults. However, the current results are highly consistent with the only other placebo-controlled study of adverse LT effects [41] in the extant literature. As in the present investigation, Volz and colleagues observed no significant differences in the side effects engendered by bright light versus dim red light. However, because these investigators utilized an outmoded light intensity by current practices in their LT condition -2500 lux, as opposed to the standard of 10,000 lux – the present study represents an important replication and extension of their work. Likewise, it stands as the first reported examination of LT side effects in a non-clinical sample.

**Figure 1:**
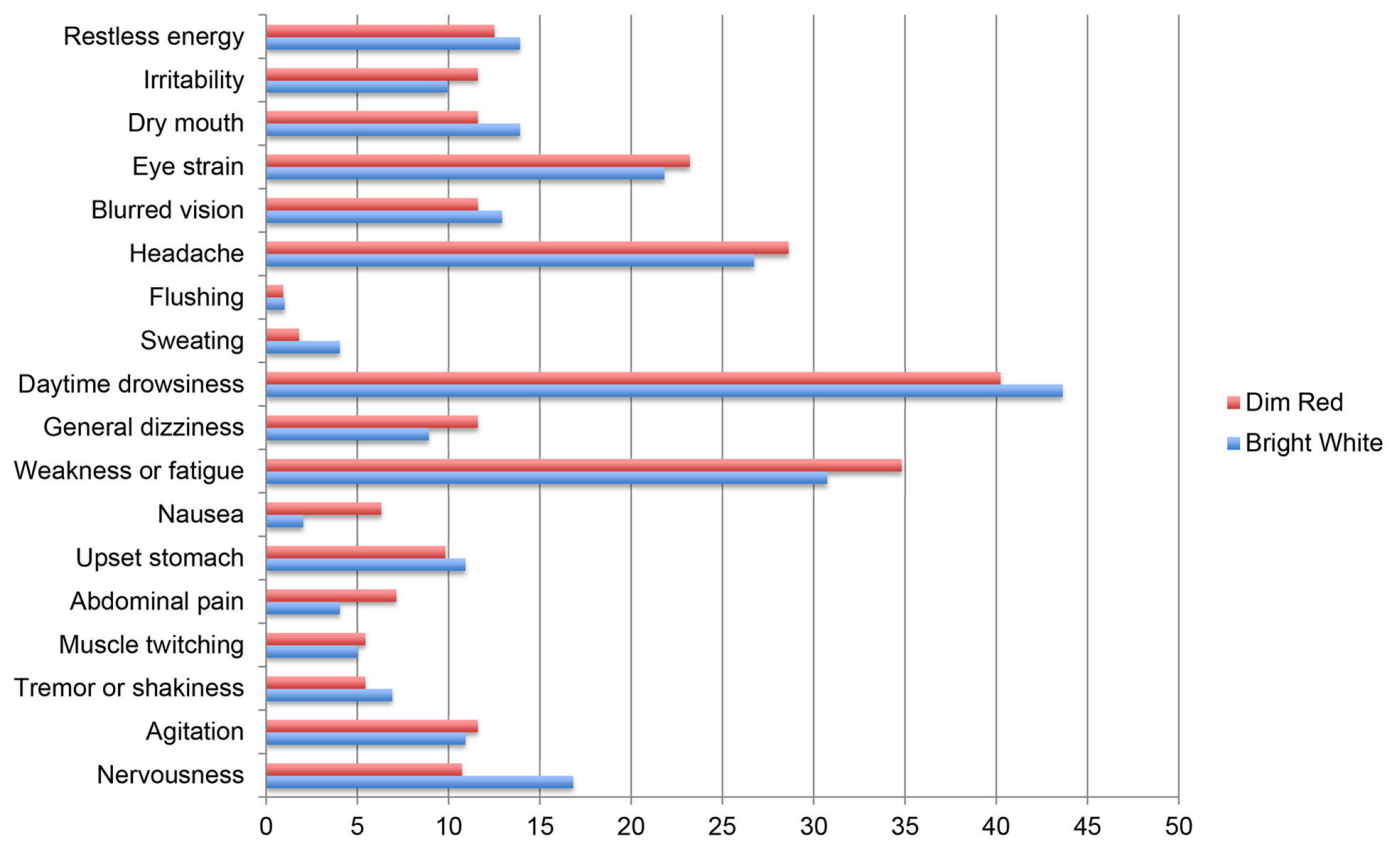
Percentage of Side Effects Reported for Dim Red and Bright White Light at Post-Treatment.

It is important to note, however, that we *did* observe modest but statistically significant increases in the occurrence of two side effects – eye strain and blurred vision – following light exposure in the present study. But these small-magnitude increases were equally likely among participants in both treatment conditions, and were thus not specific effects of LT. Specifically, at post-treatment, we observed eye strain incidence rates of 22% and 23%, respectively, in the bright light versus dim light exposure groups; likewise, we found incidence rates of 12% versus 13%, respectively, for blurred vision (Figure 1). The great majority of affected participants in both groups rated these symptoms as only “mild” in severity. While uncontrolled LT investigations have also reported such visual side effects with bright light exposure [50], the present study suggests that they are a consequence of neither the illuminance nor the color of light. In fact, they may simply reflect an artifact of the experimental task (reading) used to occupy attention during the 30-minute light exposure procedure.

Perhaps the most surprising study finding was the observation in both treatment groups of significant *decreases* in three reported side effects – nervousness, muscle twitching, and sweating – from pre-treatment to post-treatment. Rather than posit some sort of beneficial placebo-like impact of acute treatment on these three domains, we believe the finding is best attributed to mere participant habituation of the experimental setting.

The present study is characterized by a few important limitations. Notably, the generalizability of the present findings may be limited by the fact that we utilized only a single session of light exposure – a procedure that contrasts sharply with the typical administration of LT, which spans over a minimum of several days, and often over several weeks. While previous findings suggest that LT side effects tend to *diminish* over the course of repeated exposure sessions [38], we cannot rule out the possibility that some potential side effects were insufficiently triggered by a single session’s exposure. In fact, clarifying the pattern of temporal onset across potential LT side effect domains is a task that warrants the attention of future investigators in the area.

Another potential limitation lies in our use of a relatively young participant sample – a mean age of 19 – with a low average level of undesired physical symptoms at baseline (as reflected in pre-treatment responses to the study’s side effects measure). It would be desirable, therefore, to include in any attempted replication participants with a broader range of ages, including older individuals who may express greater variability in adverse side effects. Furthermore, our sample was exposed to bright light no earlier than 8 am, which contrasts with prior studies that initiate exposure earlier in the morning. However, college students are often phase shifted as indicated by our sample’s average length of sleep (less than 7 hours). Thus, we believe that the current investigation’s timing of exposure was fairly congruent for the recommended timing when adjusting for age.

## Conclusion

The principal strength of the present study lies in its use of a placebo control group, as well as a rather large sample size, in a novel examination of LT-related side effects. The current investigation also controls for the presence of any side effects present *prior to light exposure*, thereby permitting an examination of the directionality of side effect severity. Commonly, studies of side effects only examine prevalence rates *after* intervention – i.e., only the emergence or remission of symptoms rather than their increase or decrease in severity. Furthermore, the present study utilized the currently accepted dosage of LT (10,000 lux) in a non-clinical sample, thereby addressing a significant gap in the extant LT literature.

The present findings thus make a potentially significant contribution to our understanding of LT side effects, an important area of investigation in light of the increasingly frequent use of LT in both clinical and non-clinical settings. Specifically, our results indicate that the reported prevalence of acute adverse side effects in the published clinical literature may not apply fully to non-clinical populations, as no increases in headache or nausea emerged in the present investigation, in contrast with the consensus of previously published reports. Moreover, although eye strain and blurred vision increased significantly (albeit modestly) from pre-treatment to post-treatment, such increases occurred to an equivalent extent in both the bright light and control conditions; thus, they did not appear to be a function of bright light exposure, per se. Taken together, the present study results suggest that LT may be particularly well tolerated by non-clinical populations. Nevertheless, further research is needed to clarify the extent to which these results may extend beyond young, non-clinical samples, as well as the degree to which some side effects may emerge over the course of repeated LT exposure.
